# Unilateral biportal endoscopic versus microscopic transforaminal lumbar interbody fusion for lumbar degenerative disease: a retrospective study

**DOI:** 10.1186/s13018-024-04813-w

**Published:** 2024-06-01

**Authors:** Rattalerk Arunakul, Suthiya Anumas, Pattharawin Pattharanitima, Chananyu Susrivaraput, Waroot Pholsawatchai

**Affiliations:** 1 Department of Orthopaedics, Faculty of Medicine, Thammasat University, Pathumthani, 12120, Thailand; 2 Chulabhorn International College of Medicine, Thammasat University, Pathumthani, 12120, Thailand; 3 Department of Medicine, Faculty of Medicine, Thammasat University, Pathumthani, 12120, Thailand

**Keywords:** Minimally invasive surgery, Lumbar interbody fusion, Endoscopy

## Abstract

**Background:**

In the past decade, Minimally invasive transforaminal lumbar interbody fusion (MIS-TLIF) with a microscopic tubular technique has become a surgical procedure that reduces surgical-related morbidity, shortens hospital stays, and expedites early rehabilitation in the treatment of lumbar degenerative diseases (LDD). Unilateral biportal endoscopic transforaminal lumbar interbody fusion (Endo-TLIF) has emerged as a novel surgical technique. The present study aims to compare the clinical outcomes and postoperative complications of MIS-TLIF and Endo-TLIF for treating LDD.

**Methods:**

A retrospective analysis of LLD patients undergoing either Endo-TLIF or MIS-TLIF was performed. Patient demographics, operative data (operation time, estimated blood loss, length of hospitalization), and complications were recorded. The visual analog scale (VAS) score for leg and back pain and the Oswestry Disability Index (ODI) score were used to evaluate the clinical outcomes.

**Results:**

This study involved 80 patients, 56 in the MIS-TLIF group and 34 in the Endo-TLIF group. The Endo-TLIF group showed a more substantial improvement in the VAS for back pain at 3 weeks post-surgery compared to the MIS-TLIF group. However, at the 1-year mark after surgery, there were no significant differences between the groups in the mean VAS for back pain and VAS for leg pain. Interestingly, the ODI at one year demonstrated a significant improvement in the Endo-TLIF group compared to the MIS-TLIF group. Additionally, the MIS-TLIF group exhibited a shorter operative time than the Endo-TLIF group, with no notable differences in estimated blood loss, length of hospitalization, and complications between the two groups.

**Conclusion:**

Endo-TLIF and MIS-TLIF are both safe and effective for LDD. In surgical decision-making, clinicians may consider nuances revealed in this study, such as lower early postoperative back pain with Endo-TLIF and shorter operative time with MIS-TLIF.

## Introduction

Lumbar degenerative disease (LLD) is a common condition resulting from degenerative changes in the spine, causing chronic pain, instability, and subsequent neurological impairments that lead to a loss of daily function and quality of life. Transforaminal lumbar interbody fusion (TLIF) has been widely accepted as the surgical treatment of choice for decompressing neural elements and stabilizing bony structures [1]. As the population continues to age, the demand for spinal fusion surgery is likely to increase, leading to a higher occurrence of postoperative complications in elderly patients with comorbidities [2–4].

Conventional open TLIF stands as a viable choice for addressing degenerative lumbar spinal disease. Nevertheless, it poses the risk of various complications, including the potential for back muscle atrophy and the development of postlaminectomy syndrome. These complications arise from the extensive dissection and retraction of muscles associated with the procedure. [5, 6] On the other hand, Minimal invasive transforaminal lumbar interbody fusion (MIS-TLIF) offers favorable outcomes while minimizing the risk of complications by utilizing a tubular retractor and a surgical microscope, making it a preferable alternative [1, 7, 8]. Moreover, several studies have reported statistically significant lower pain levels, faster recovery, and shorter hospital stays when comparing MIS-TLIF to conventional TLIF [1, 9–14].

In the 2000s, endoscopic techniques were introduced in TLIF procedures, with pioneering surgeons reporting successful endoscopic decompression of foraminal pathology [1]. Chen et al. reported a statistically significant improvement in the Oswestry Disability Index (ODI) score in the endoscopic group compared to microscopic discectomy for lumbar disc herniation in a randomized controlled trial [15, 16]. A recent retrospective cohort study demonstrated statistically significant lower blood loss and hospital stay in the endoscopic TLIF group compared to MIS-TLIF [17].

Both MIS-TLIF and Endoscopic Transforaminal Lumbar Interbody Fusion (Endo-TLIF) have shown excellent outcomes while minimizing associated risks and have proven to be promising techniques for patients with a lumbar degenerative disease requiring surgical intervention. Given the limited existing research on both MIS-TLIF and Endo-TLIF, we initiated a retrospective cohort study to assess and compare their respective clinical outcomes.

## Materials and methods

### Patient population and grouping

This retrospective study enrolled 90 patients (22 males, 68 females) diagnosed with Lumbar degenerative disease who underwent either Endo-TLIF or MIS-TLIF at our department between January 2012 and January 2023. The study was conducted in compliance with the guidelines of the Declaration of Helsinki and received approval from Thammasat University's ethics committee. Among the patients, 34 underwent Endo-TLIF, while the remaining 56 underwent MIS-TLIF.

The inclusion criteria for this study encompassed patients aged between 20 and 75 years who had undergone single or two-segment-level transforaminal lumbar interbody fusion and experienced radiating pain in the lower extremities (visual analog scale (VAS) score ≥ 4) and/or neurogenic intermittent claudication. Additionally, eligible participants exhibited definite lumbar spinal stenosis with or without low-grade degenerative spondylolisthesis (grade ≤ 2), low-grade isthmic spondylolisthesis (grade ≤ 2), and segmental instability (anterior translation (> 3 mm) and/or increasing segmental sagittal motion (> 15 °)) on plain standing radiographs and magnetic resonance imaging (MRI). Exclusion criteria comprised patients with previous lumbar spine surgery or revision, high-grade spondylolisthesis (Mayerding > 2), those diagnosed or suspected to have underlying or ongoing diseases such as spondylodiscitis, ankylosing spondylitis, spinal neoplasm, spinal metastasis, and traumatic spine injury, as well as individuals diagnosed with cognitive or psychological disorders [4, 18].

Each patient meeting the inclusion criteria underwent either Endo-TLIF or MIS-TLIF under general anesthesia. The surgical approach was selected through preoperative discussions, considering various patient factors and surgeon preferences.

### Surgical techniques

#### Minimally invasive transforaminal lumbar interbody fusion (MIS-TLIF)

The posteroanterior and lateral fluoroscopy techniques were employed to accurately locate the pedicles at the surgical level. The Wiltse approach [19], involving a paramedian skin incision, was utilized. A Quadrant tubular dilator was inserted to achieve unilateral facet exposure, and ipsilateral facetectomy was performed to visualize the transforaminal disc space. Laminectomy and decompression of the lateral recess were conducted to alleviate spinal canal compression. Additionally, the tubular retractor could be angled inward to enhance decompression for central canal stenosis and the contralateral side. The ligamentum flavum was appropriately removed to expose the ipsilateral traversing and exiting nerve roots. Standard discectomy and removal of the endplates were performed to facilitate the insertion of an intervertebral cage.

Autogenous and allogeneic bone grafts were placed anteriorly and contralateral to the annulotomy, followed by inserting an intervertebral cage filled with a combination of autogenous and allogeneic bone grafts. Moreover, unilateral pedicle screws were inserted on the same side as the approach, while contralateral pedicle screws were placed through a separate incision. Correctly sized rods were subfascially tunnelled through the paramedian incisions. The incisions were thoroughly irrigated and closed layer by layer, and drains were placed to prevent fluid accumulation.

#### Unilateral biportal endoscopic transforaminal lumbar interbody fusion (Endo-TLIF)

**Fig. 1:**
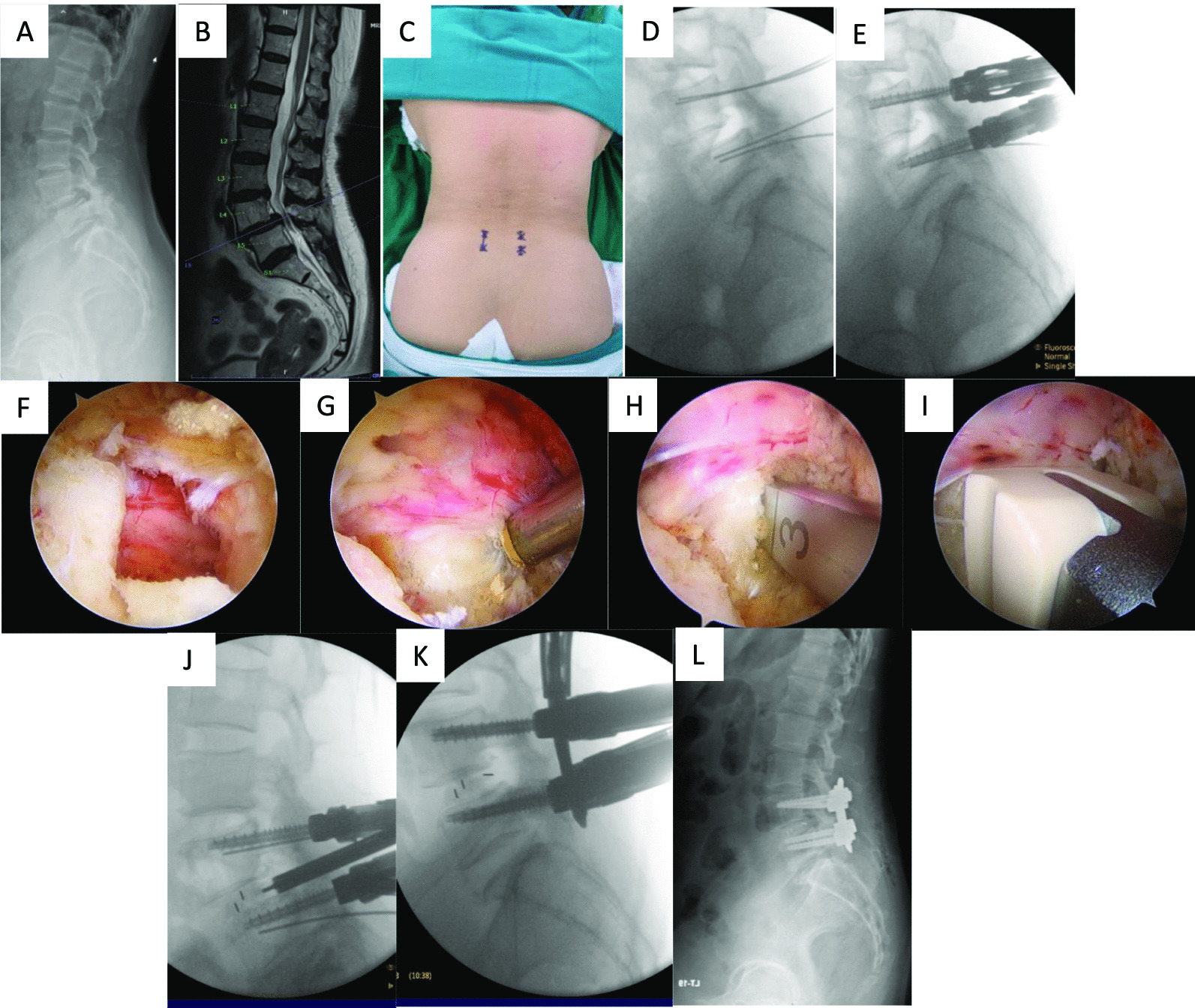
Case demonstration of Endo-TLIF **A**: Preoperative plain radiograph revealed low-grade spondylolisthesis of L4/L5. **B**: MRI revealed spinal stenosis at L4/L5. **C**: Skin incision marked. **D**: Intraoperative fluoroscopy revealed the guide wire was inserted into the upper and lower pedicle. **E**: Contralateral pedicle screw was inserted. **F**: Intraoperative endoscope revealed the laminectomy was done, and the ligamentum flavum was removed. **G**: The intervertebral disc was identified. H: Intervertebral disc was removed by disc shaver. **I**: The intervertebral cage was inserted into the intervertebral disc space. **J**: The position of the intervertebral cage was con-firmed with fluoroscopy. **K**: The pedicle screws were connected by a rod. **L**: Postoperative plain radiograph after L4/L5 Endo-TLIF

Under fluoroscopic guidance, separate longitudinal skin incisions were marked 1 cm lateral to the upper and lower pedicles. A standard sterile preparation was carried out before making the marked skin incisions. Subsequently, a Pak needle was inserted into the upper and lower pedicles, and a guide wire was then placed to prepare for pedicle screw insertion. The pedicle screw on the contralateral side was inserted before the portal was created (Fig. 1).

At the pedicle screw insertion point, The portal was carefully created to allow for adequate instrument access through the superficial fascia, ensuring sufficient saline flow. A muscle detacher was employed to make space for water flow through a portion of the proximal lamina and the interlaminar space. In the left-sided approach, the upper portal served as the viewing portal, while the lower portal functioned as the working portal. An arthroscopic irrigation system was employed in the procedure, enabling saline irrigation fluid to drain from the viewing portal to the working portal.

The surgical technique followed a similar approach to MIS-TLIF. Ipsilateral laminectomy was performed using a burr, Kerrison punch, and osteotome. Subsequently, contralateral sublaminar decompression was carried out. Harvesting of autologous bone involved performing unilateral facetectomy using osteotomes. After removing the inferior articular process, the superior articular process was carefully removed to create a space between the exiting and traversing nerve roots. After completing the ipsilateral and contralateral decompressions and facetectomies, the ligamentum flavum covering the dura and nerve root was removed.

An incision was made on the disc using a knife, and a discectomy was performed using pituitary forceps, a curette, and a disc shaver. The arthroscope was inserted into the disc space to monitor the area, and the cartilaginous endplate was meticulously removed using a curette to expose the subchondral bone. Allogenic bone chips and autologous bone harvested from the lamina and facet were filled into the intervertebral cage. A cage was vertically inserted with guidance from fluoroscopy and arthroscopy.

The percutaneous pedicle screws on the ipsilateral side were inserted through the previously prepared guide wire. The pedicle screws were connected by percutaneously inserting a rod. A drain catheter was also inserted to facilitate the drainage of potential epidural hematoma or small bony debris, concluding the operation.

### Statistical analyses

According to Kim et al. [20], the mean (SD) Visual Analog Scale (VAS) scores for back pain at two weeks postoperative of Endo-TLIF and MIS-TLIF were 3.1 (1.0) and 4.2 (1.6), respectively. The sample size was determined using the 16th version of STATA with a two-sample mean test (Power 90% and alpha = 0.05), resulting in 33 patients in each group, totaling N = 66. The primary objective of this study is to compare the clinical efficacy of Endo-TLIF and MIS-TLIF for lumbar degenerative disease, utilizing the VAS to assess clinical outcomes. VAS scores for back and leg pain were collected at baseline before the surgery and at 3, 6 weeks, 3, 6, and 12 months post-surgery. Additionally, the outcomes of each group are being evaluated using the Oswestry Disability Index (ODI) [14], and secondary objectives include monitoring complications such as wound infection and deep venous thromboembolism. Independent t-tests were employed to compare normal continuous variables between the two groups, while Fisher's exact test was used for categorical variables. A *p*-value of ≤ 0.05 was considered statistically significant.

## Results

### Demographic data

**Fig. 2:**
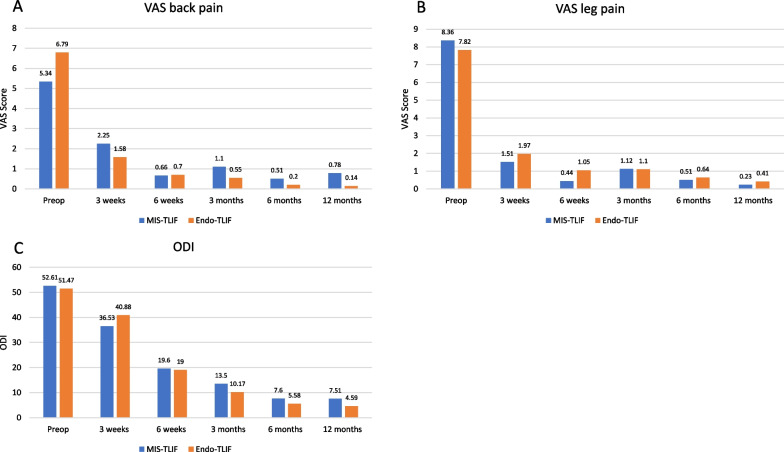
Clinical outcomes during follow-up (preoperative, postoperative 3 weeks, postoperative 6 weeks, postoperative 3 months, postoperative 6 months, postoperative months): **A** VAS of back pain. **B** VAS of leg pain. **C** ODI. ODI; indicates Oswestry Disability Index, VAS; Visual Analogue Scale

The demographic and clinical attributes of individuals undergoing distinct lumbar fusion techniques were compared. Regarding gender distribution, both groups predominantly consisted of females, constituting 76.79% in the MIS-TLIF and 73.53% in the Endo-TLIF group. The respective male percentages were 23.21% and 26.41%, showing no statistically significant difference (*P*-value = 0.8) (Table 1) (Fig. 2).

**Table 1:** Comparison of demographic data between the two groups

Data	MIS-TLIF (56)	Endo-TLIF (34)	*P*-value
Sex			
Male	13 (23.21%)	9 (26.41%)	0.8^2^
Female	43 (76.79%)	25(73.53%)	
Age (years)	62.84 (13.80)	61.26 (9.28)	0.24^1^
Weight (kg)	64.05 (10.74)	69.83 (10.81)	0.006^1,o^
Height (cm.)	157.46 (7.46)	156.62 (5.63)	0.77^1^
BMI (kg/m^2^)	13 (23.21)	28.55 (4.29)	< 0.001^1,o^
Diagnosis			
Spondylolisthesis	32 (57.14%)	25 (73.53%)	0.17^2^
Spinal stenosis	24 (42.86%)	9 (26.47%)	
Level			
1 level	45 (80.36%)	26 (76.47%)	0.79^2^
2 levels	11 (19.64%)	8 (23.53%)	
Preoperative symptoms			
Back pain	40 (71.43%)	28 (82.35%)	0.31^1^
Radicular pain	54 (96.43%)	28 (82.35%)	0.06^1^
Numbness	28 (50%)	23 (67.65%)	0.13^1^
Motor weakness	6 (10.71%)	7 (20.59%)	0.23^1^
Preoperative score			
VAS back pain	5.34 (3.76)	6.79 (3.26)	0.08^1^
VAS leg pain	8.36 (6.21)	7.82 (2.18)	0.99^1^
ODI	52.61(16.33)	51.47 (11.28)	0.47^1^

^1^Calculated by T-test [Value are presented as Mean (SD)], ^2^Calculated by Fisher exact test [Value are presented as n(%)], ^o^Statistical significant

Upon examining age and anthropometric measures, no significant distinctions emerged in mean age, height, or diagnoses of Spondylolisthesis and Spinal Stenosis. However, the MIS-TLIF group exhibited a significantly lower mean weight (64.05 kg) than the Endo-TLIF group (69.83 kg), with a *P*-value of 0.006. Correspondingly, the Body Mass Index (BMI) was notably lower in the MIS-TLIF group (25.79 kg/m^2^) compared to the Endo-TLIF group (28.55 kg/m^2^), with statistical significance (*P*-value < 0.001).

The distribution of treated levels, categorized into 1 and 2 levels, displayed no significant differences between the groups. Variations in preoperative symptoms were observed, and no statistical significance was reached for back pain, radicular pain, claudication, and weakness.

Visual Analog Scale (VAS) scores for back pain and leg pain showed no statistically significant differences, except for a trend toward significance in VAS back pain (*P*-value = 0.08), indicating a slightly lower mean score in the MIS-TLIF group. The analysis highlights generally comparable demographic and clinical characteristics between MIS-TLIF and Endo-TLIF groups, with notable differences in weight and BMI. The findings underscore that radicular pain did not reach statistical significance between the two lumbar fusion procedures.

### VAS and ODI

The comprehensive comparison of Visual Analog Scale (VAS) scores for back and leg pain, along with Oswestry Disability Index (ODI) scores, between two lumbar fusion techniques (MIS-TLIF and Endo-TLIF) spans various postoperative intervals: three weeks, six weeks, three months, six months, and twelve months (Table 2).

**Table 2:** Comparison of VAS and ODI Scores data between the two groups

Outcomes	MIS-TLIF (56)	Endo-TLIF (34)	*P*-value ^1^
VAS back pain			
3 weeks postoperative	2.25(1.45)	1.58(1.41)	0.04^o^
6 weeks postoperative	0.66(1.56)	0.7(1.38)	0.49
3 months postoperative	1.1(6.6)	0.55(1.70)	0.57
6 months postoperative	0.51(1.34)	0.2(1.03)	0.16
12 months postoperative	0.78(1.8)	0.14(0.7)	0.06
VAS leg pain			
3 weeks postoperative	1.51(1.96)	1.97(1.71)	0.13
6 weeks postoperative	0.44(1.34)	1.05(2.15)	0.16
3 months postoperative	1.12(4.26)	1.10(6.69)	0.84
6 months postoperative	0.51(1.51)	0.64(1.90)	0.76
12 months postoperative	0.23(0.95)	0.41(1.43)	0.73
ODI			
3 weeks postoperative	36.53(16.20)	40.88(10.71)	0.13
6 weeks postoperative	19.6(12.18)	19(14.69)	0.55
3 months postoperative	13.5(12.67)	10.17(11.57)	0.12
6 months postoperative	7.6(9.25)	5.58(9.5)	0.17
12 months post-op	7.32(10.81)	2.82(7.58)	< 0.001^o^

^1^Calculated by T-test [Mean(SD)], ^o^Statistical significant

Regarding VAS back pain, a statistically significant difference favoring the Endo-TLIF group is observed at three weeks postoperatively (*P*-value = 0.04). However, no significant differences are found at subsequent intervals, with *P*-values ranging from 0.06 to 0.57 at 12 months postoperatively. No statistically significant differences are identified for VAS leg pain at any evaluated time point, with *P*-values ranging from 0.13 to 0.84. The scores remain comparable between the MIS-TLIF and Endo-TLIF groups throughout the postoperative period.

Regarding ODI scores assessing functional disability, no statistically significant differences are observed at three weeks, six weeks, three months, and six months postoperatively. However, a significant difference is noted at 12 months postoperatively (*P*-value =  < 0.001), suggesting a potential advantage for the Endo-TLIF group with significantly lower mean scores. These findings indicate that while both procedures demonstrate comparable outcomes in leg pain and early functional disability, Endo-TLIF may offer advantages in terms of early postoperative back pain.

### Surgical technique-related outcome and complication

The comparative analysis of surgical technique-related outcomes between MIS-TLIF and Endo-TLIF includes assessing operation time, estimated blood loss (measured by Hematocrit drop), and length of hospitalization (Table 3).

**Table 3:** Comparison of surgical technique related outcomes between the two groups

Outcomes	MIS-TLIF (56)	Endo-TLIF (34)	*P*-value^1^
Operation time (min)	237.33 (50.65)	282.76 (58.82)	< 0.001^o^
Estimated blood loss (hematocrit drop)	3.95 (1.59)	3.78 (1.88)	0.39
Length of hospitalization (days)	4.23 (2.01)	4.00 (0.70)	0.16

^1^Calculated by T-test [Mean(SD)], ^o^ Statistical significant

Regarding operation time, the MIS-TLIF group exhibited a significantly shorter duration, with a mean of 237.33 min, compared to the Endo-TLIF group, which had a mean of 282.76 min (*P*-value < 0.001). This indicates a notable time advantage associated with the MIS-TLIF procedure. The estimated blood loss, as indicated by the hematocrit drop, showed no statistically significant difference between the two groups. The mean values were 3.95 for MIS-TLIF and 3.78 for Endo-TLIF, with a *P*-value of 0.39. Similarly, the length of hospitalization demonstrated no significant disparity between the groups. The MIS-TLIF group had a mean hospital stay of 4.23 days, while the Endo-TLIF group had a mean of 4.00 days (*P*-value = 0.16).

Our analysis revealed a single case within the MIS-TLIF group experiencing an epidural hematoma and subsequent cauda equina syndrome on postoperative day 2. This condition was effectively addressed through hematoma removal, and a reassuring outcome was observed during the 6-month follow-up, with the patient's neurological status returning to normal. Additionally, another case of postoperative wound infection surfaced in the MIS-TLIF group at two weeks postoperative. This infection was successfully managed through one-time surgical debridement and intravenous antibiotics. In the Endo-TLIF group, there was one case of inadequate decompression, which was corrected with a reoperation involving decompression. Importantly, we observed no instances of dura tear or implant failure in our series after the 12-month follow-up period (Table 4).

**Table 4:** Comparison of complications between the two groups

Complication	MIS-TLIF (56)	Endo-TLIF (34)
Hematoma	1(1.78)	0
Incomplete decompression	0	1(2.94)
Surgical site infection	1(1.78)	0
Dura tear	0	0
Implant failure	0	0

Number of patients (%)

## Discussion

In this retrospective cohort study, we have demonstrated that Endo-TLIF delivers comparable clinical outcomes to MIS-TLIF at the 12-month follow-up. Endo-TLIF and MIS-TLIF procedures effectively reduced pain, as measured by VAS, and decreased disability, as indicated by the ODI scores after surgery. However, a significant difference was observed between the two groups in VAS for back pain at three weeks postoperatively. However, no difference was noted at other points in time during follow-up. Furthermore, the ODI at 12 months showed a significant difference in the Endo-TLIF group, with no significant differences at other follow-up intervals. Both techniques demonstrated comparable efficacy in reducing leg pain and improving functional disability.

Since the introduction of the TLIF technique, it has gained widespread acceptance as a viable alternative to PLIF. In contrast to PLIF, TLIF offers the advantage of decom-pressing the foramen and facilitating the restoration of interbody height. Notably, TLIF allows cage insertion without nerve retraction, anticipating a reduction in intraoperative bleeding. [21–25] As the twenty-first century unfolded, the Minimally invasive TLIF was developed to respond to the potential for significant muscle injury associated with traditional open surgery. This technique aims to reduce soft tissue and muscle damage, enabling surgical procedures with minimal impact on the middle and contralateral spinal structures through unilateral access. The evolution of minimally invasive surgery, incorporating endoscopes, became possible due to advancements in optical technologies and the introduction of specialized instruments.

Zhang et al. [19] conducted a comparative study between Endo-TLIF and MIS-TLIF, revealing that VAS back pain and JOA scores significantly improved within one week, with no notable differences in subsequent intervals. In another investigation, Ju-Eun Kim et al. [20] reviewed patients undergoing MIS-TLIF and Endo-TLIF for single-level lumbar disease. They reported a significant improvement in VAS scores for back pain at two weeks and two months postoperatively, with no difference at other points. Additionally, Min-Seok Kang et al. [26] performed a retrospective review of patients undergoing Endo-TLIF and MIS-TLIF, demonstrating significant improvements in VAS back pain and SF-36 scores within one month, with no differences at other follow-up times.

Our results indicate that Endo-TLIF reduces early postoperative back pain (3 weeks postoperative), supporting previous study findings. Utilizing a minimally invasive approach is associated with reduced postoperative pain and improved clinical outcomes, as highlighted in various studies [27, 28]. The process of intraoperative dissection and retraction of paraspinal muscles during traditional approaches may lead to atrophy and denervation, potentially contributing to increased postoperative pain. Research by Kawaguchi and colleagues has specifically addressed the impact of the retractor blade's pressure and duration on paraspinal muscles [29].

**Fig. 3:**
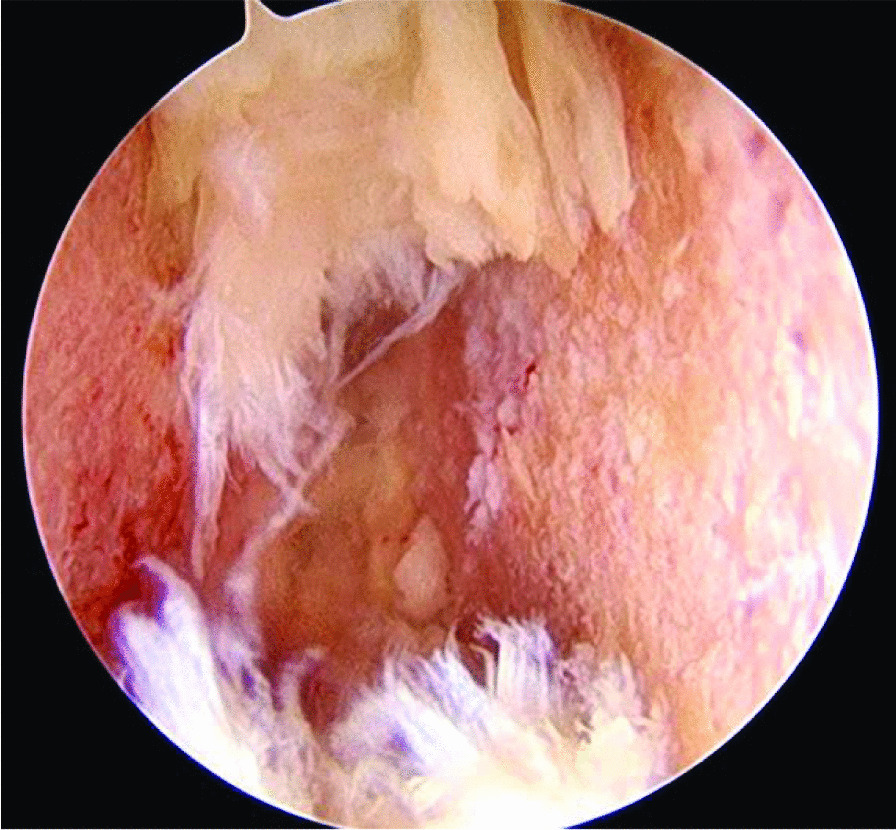
The intradiscal view under endoscopy reveals complete cartilage endplate removal and proper bone graft bed preparation

Our study revealed a significant decrease in disability scores 12 months postoperatively in the Endo-TLIF group. Potential factors contributing to this outcome include: (1) The use of a tubular retractor in MIS-TLIF may induce surgical trauma to the skin and muscle, potentially leading to postoperative skin and muscle necrosis, as well as long-term scar healing issues. In contrast to MIS-TLIF, Endo-TLIF eliminates the need for placing a tubular retractor between paraspinal muscles, thereby minimizing direct ischemic damage. (2) The importance of proper endplate preparation in facilitating spinal fusion and stability. In MIS-TLIF, endplate preparation relies on tactile sensation, making direct observation difficult and increasing the likelihood of inadequate cartilage removal or bony endplate damage. Conversely, in Endo-TLIF, complete cartilage endplate removal and bone graft bed preparation can be achieved through magnified visualization under endoscopy, creating an optimal environment for bone graft fusion (Fig. 3). Previous literature, such as the review by Wang and colleagues, supports these findings, demonstrating that the interbody fusion rate, as classified by Mannion's fusion classification, was significantly better in endoscopic lumbar interbody fusion compared to MIS-TLIF at six months and one year postoperatively. Unfortunately, we did not focus on or report the fusion rate in this study.

In our study, a higher proportion of patients with greater body weight in the Endo-TLIF group compared to the MIS-TLIF group. The increased BMI among patients in the Endo-TLIF group was coincidental rather than biased patient selection. Dissecting posterolaterally to identify the lamina and facet joint through thick soft tissue and paraspinal muscle in obese patients presents a significant challenge. However, this challenge is effectively mitigated through the utilization of endoscopy. The procedure incorporates an endoscope, which enhances magnification and enables more precise work, allowing direct and complete surgical site visualisation. This grants the surgeon an optimized view of anatomical structures without a loss of sight caused by the larger depth in obese patients. Ultimately, both groups (Endo-TLIF and MIS-TLIF) demonstrated similar results regarding postoperative pain and functional scores.

Leyendecker et al. investigated the influence of obesity on full endoscopic spine surgery outcomes through a comparative analysis between obese and non-obese patients. Their findings revealed that obese patients exhibited accelerated early recovery, characterized by significantly greater improvements in Oswestry Disability Index (ODI) and leg pain at seven days post-surgery. However, no disparity in improvement between the groups was observed at the 90-day mark following surgery [30]. Moreover, Xu Shen et al. [31] compared percutaneous endoscopic posterior lumbar interbody fusion (Endo-LIF) and TLIF in treating obese patients with LDD; their findings revealed that the Endo-LIF group experienced significantly less blood loss, a shorter time to postoperative ambulation, fewer complications, and shorter hospitalization days, despite a longer operation time. They also noted that the Endo-LIF group had greater ease in exposing soft tissue and facilitating assistance. Their results suggest that Endo-TLIF remains a viable option for patients with higher body weight or obesity, offering easier implementation, smaller incisions, and improved visualization.

We observed that Endo-TLIF required a significantly longer operation time compared to MIS-TLIF. This may be attributed to the fact that Endo-TLIF is a relatively newer technique, requiring a longer learning curve for surgeons to become proficient. Consequently, the surgical procedure requires more time for tasks such as creating sufficient space for the operation, identifying surgical landmarks, and conducting proper decompression.

Despite the longer operation time, Endo-TLIF demonstrated a favorable outcome with lower perioperative blood loss compared to MIS-TLIF, although the difference was not statistically significant. Previous literature [26] has indicated that Endo-TLIF significantly reduces perioperative blood loss due to decreased muscle dissection and a smaller skin incision. However, this was not observed in our study, and it may be attributed to the longer operative time in our research, which could potentially increase bleeding during the procedure.

Nevertheless, no significant differences between Endo-TLIF and MIS-TLIF concerning the hospitalization duration and complications were observed. These results suggest that an extended operation time, often considered a risk factor for postoperative complications and unfavorable outcomes, does not apply to Endo-TLIF.

Various techniques for endoscopic lumbar interbody fusion are described in the literature, including full-endoscopic lumbar interbody fusion, full-endoscopic trans-kambin triangle lumbar interbody fusion, and biportal endoscopic lumbar interbody fusion. The unilateral biportal endoscopic TLIF is similar to MIS TLIF surgery, involving direct decompressive techniques such as ipsilateral laminotomy and total facetectomy in the posterolateral approach. Although full-endoscopic TLIF through the trans-Kambin approach is less invasive than the posterolateral approach, it poses the disadvantage of exiting nerve root injury. Since a cage is inserted through Kambin's triangle, there might be a high possibility of exiting nerve root injury during insertion. Direct decompression of the contralateral nerve root and endplate preparation may also be limited in the trans-Kambin approach [32].

Uniportal endoscopic lumbar interbody fusion is developed using the posterolateral approach. In this technique, endoscopy and instruments utilize the same enclosed space within the working cannula. However, inserting a cage larger than the diameter of the working cannula obstructs the endoscopic view. Consequently, the cage must be inserted under C-arm guidance without endoscopic observation. This may lead to complications including cage malposition, vertebral endplate injury, and dural tear. In contrast, Biportal endoscopic surgery allows surgeons to utilize both hands by employing separate endoscopic and working portals. This separation facilitates clear visualization during cage insertion procedures, minimizing the likelihood of complications [33].

The present study has several limitations that warrant acknowledgement. We did not conduct blood exams to evaluate the inflammatory process or muscle injury between the two procedures, such as creatinine protein kinase (CPK) and erythrocyte sedimentation rate (ESR). These are not routine labs in our protocol. It is important to note that this study was retrospective in nature and lacked randomization, making it a non-randomized controlled cohort study. There is a possibility of selection bias since the decision to perform microscopic or endoscopic was based on the individual surgeon's judgment. To obtain more robust conclusions, it is recommended to conduct further prospective, randomized, controlled studies with larger sample sizes and longer follow-up durations to determine the optimal surgical approach for patients with lumbar degenerative disease.

## Conclusions

Our retrospective cohort study confirms that Endo-TLIF is comparable to MIS-TLIF in clinical outcomes at the 12-month follow-up, effectively reducing both pain and disability. Both procedures demonstrate similar effectiveness in managing leg pain and improving functional outcomes.

The acceptance of TLIF as a minimally invasive alternative has grown, with Endo-TLIF addressing concerns about muscle injury associated with traditional open surgery. Our findings align with previous studies, supporting Endo-TLIF's effectiveness in reducing early postoperative back pain. Despite a longer operation time for Endo-TLIF, favorable outcomes are observed, and challenging assumptions about prolonged procedures lead to complications. The study underscores Endo-TLIF as a viable option, especially for patients with higher body weight. While acknowledging study limitations, such as its retrospective nature, further prospective, randomized studies are essential to validate the optimal surgical approach for lumbar degenerative disease. Overall, our research provides valuable insights into the evolving landscape of spinal surgery, emphasizing the potential benefits of Endo-TLIF in enhancing postoperative outcomes.
